# Giving tranexamic acid to reduce surgical bleeding in sub-Saharan Africa: an economic evaluation

**DOI:** 10.1186/1478-7547-8-1

**Published:** 2010-02-17

**Authors:** Carla Guerriero, John Cairns, Sudha Jayaraman, Ian Roberts, Pablo Perel, Haleema Shakur

**Affiliations:** 1 Department of Public Health and Policy, London School of Hygiene & Tropical Medicine, London, UK; 2 Department of Surgery and Global Health Sciences, University of California at San Francisco, San Francisco, California; 3 Department of Epidemiology and Population Health, London School of Hygiene & Tropical Medicine, London, UK

## Abstract

**Background:**

The identification of safe and effective alternatives to blood transfusion is a public health priority. In sub-Saharan Africa, blood shortage is a cause of mortality and morbidity. Blood transfusion can also transmit viral infections. Giving tranexamic acid (TXA) to bleeding surgical patients has been shown to reduce both the number of blood transfusions and the volume of blood transfused. The objective of this study is to investigate whether routinely administering TXA to bleeding elective surgical patients is cost effective by both averting deaths occurring from the shortage of blood, and by preventing infections from blood transfusions.

**Methods:**

A decision tree was constructed to evaluate the cost-effectiveness of providing TXA compared with no TXA in patients with surgical bleeding in four African countries with different human immunodeficiency virus (HIV) prevalence and blood donation rates (Kenya, South Africa, Tanzania and Botswana). The principal outcome measures were cost per life saved and cost per infection averted (HIV, Hepatitis B, Hepatitis C) averted in 2007 International dollars ($). The probability of receiving a blood transfusion with and without TXA and the risk of blood borne viral infection were estimated. The impact of uncertainty in model parameters was explored using one-way deterministic sensitivity analyses. Probabilistic sensitivity analysis was performed using Monte Carlo simulation.

**Results:**

The incremental cost per life saved is $87 for Kenya and $93 for Tanzania. In Botswana and South Africa, TXA administration is not life saving but is highly cost saving since fewer units of blood are transfused. Further, in Botswana the administration of TXA averts one case of HIV and four cases of Hepatitis B (HBV) per 1,000 surgical patients. In South Africa, one case of HBV is averted per 1,000 surgical patients. Probabilistic sensitivity analyses confirmed the robustness of the model.

**Conclusion:**

An economic argument can be made for giving TXA to bleeding elective surgical patients. In countries where there is a blood shortage, TXA would be a cost effective way to reduce mortality. In countries where there is no blood shortage, TXA would reduce healthcare costs and avert blood borne infections.

## Background

**Figure 1:**
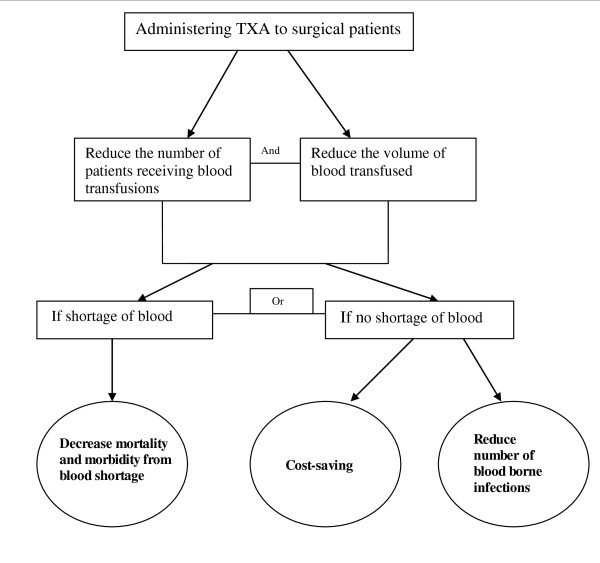
**Possible effects of administering TXA**.

The risks and costs associated with blood transfusions have increased interest in the identification of safer and cheaper alternatives. Blood sparing interventions are particularly important in sub-Saharan African countries due to the high prevalence of blood borne viral infections and blood shortages. In the African region, an average of 5 units of blood per 1000 population are donated each year compared to between 30 and 60 units in high income countries [1,2]. It has been estimated that about 150,000 women die each year during pregnancy or soon after delivery because of a shortage of blood for transfusion [3]. Even when blood is available, it can transmit potentially fatal viral infections. It has been estimated that in the African region, 99% of blood is screened for HIV, 95% for HBV and 96% for HCV [2]. The administration of the antifibrinolytic agent tranexamic acid (TXA) could be a cost effective way to reduce the need for blood transfusion. A recent systematic review of randomised controlled trials showed that the administration of TXA to elective surgical patients reduces the number of transfusions by one third and the volume of blood required per transfusion by one unit [4]. Ongoing studies are also being conducted to investigate the effectiveness of administering TXA in cases of trauma and women with post partum haemorrhage [5]. In countries with blood shortages, the administration of TXA could increase the supply of blood for those who need it. On the other hand, where blood is readily available the administration of TXA could, by reducing the need for transfusion, decrease the risk of life threatening blood borne infections and reduce costs since fewer units of blood would need to be given (Figure 1).

This suggests that the use of TXA may have benefits in sub-Saharan Africa, where resource constraints argue for cost-effective alternatives to using blood products. Nevertheless, a comprehensive literature review failed to find any studies investigating the cost-effectiveness of giving antifibrinolytic agents among elective surgical patients in developing countries.

This study uses a decision analytic model to evaluate the cost-effectiveness of using TXA to reduce the need for blood transfusion, thus potentially reducing mortality from blood shortages and preventing blood borne viral infections in four African countries.

## Methods

### The settings

Four African countries were selected to represent a range of blood donation rates and HIV seroprevalence among blood donors (Table 1). South Africa has the highest blood donation rate at 17 units of blood donated per 1,000 inhabitants per year, whereas in Tanzania fewer than 3 units of blood are donated per 1,000 inhabitants per year (Table 1) [6]. Kenya and South Africa have a low HIV prevalence among the donor population (1.2%, and <0.1% respectively) whereas the prevalence is higher in Tanzania and Botswana (2.8% and 2.1%)[6].

**Table 1:** Donation rate and HIV prevalence by country^a^

Country	**Donation rate**^**b**^	Percentage of blood collections reactive to HIV
Botswana	11.6	2.1
South Africa	17.0	0.1
Tanzania	2.7	2.9
Kenya	3.3	1.2

**a **Source data[6]

**b **Donation rate per 1,000 inhabitants per year (2007 values)

### Model

**Figure 2:**
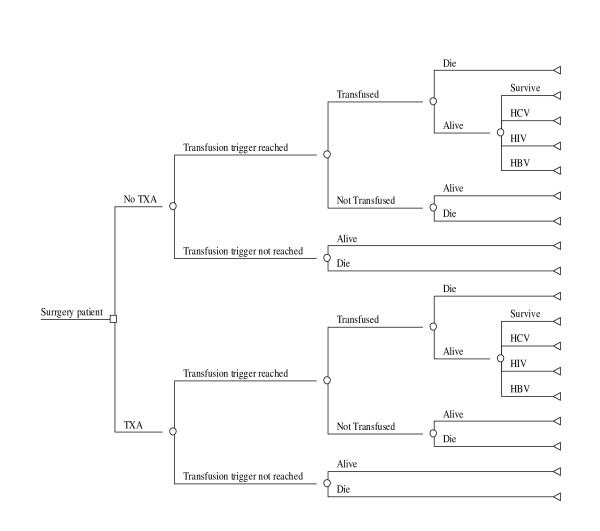
**Decision tree for Tranexamic acid administration in surgical setting**.

A decision-analytic model was developed in DataTM PRO (TreeAge software Inc., MA, USA) as shown in Figure 2. Two costs are considered in the economic analysis: the cost of blood transfusion and the cost of TXA. The analysis did not include indirect costs such as wages and productivity losses due to illness and death. The model consists of two different strategies: routinely giving TXA to surgical patients and not giving TXA [7]. The structure of the two strategies is identical, but the associated probabilities and payoffs differ. The decision model starts with the choice between administering or not administering TXA to a hypothetical cohort of 1,000 bleeding surgical patients. Whichever strategy is chosen the patient can reach the transfusion trigger and require a blood transfusion or can be healthy without requiring a blood transfusion (transfusion trigger not reached). If the patient is transfused he/she can remain healthy, can be infected (HIV, HBV, or HCV) or can die. If a patient did not receive a clinically indicated blood transfusion because it was not available then he/she has a higher probability of dying. For simplicity, it is assumed that a patient cannot be infected by more than one viral infection. The outcomes considered in the decision tree are: deaths of patients who could not receive blood transfusions due to blood shortage and the number of HIV, HCV and HBV infections.

Data on probabilities and costs are required in order to populate the decision model. Probabilities were estimated from published studies and from simple mathematical models reported in the following text. All the parameters of the model, data sources and values used are presented in Tables 1, 2 and 3 and are discussed in greater detail in the 'Cost' and 'Probability' sections. Because the costs and consequences included are within a year of treatment, no discounting is required. One-way deterministic sensitivity analysis was performed in order to estimate the impact of parameter variation on the incremental cost-effectiveness ratio. In addition, a probabilistic sensitivity analysis was also undertaken in which all utilisation and outcome variables were varied.

**Table 2:** Probabilities used to populate the model

Parameters		NO TXA	TXA
**Probability of reaching the transfusion trigger**		0.66 (0-1)	0.40 (0-0.66)
**Probability of being transfused**	Botswana	1	1
	Kenya	0.33	0.86
	Tanzania	0.27	0.70
	South Africa	1	1
**Probability of death for a surgical patient in Africa**		0.06 (0.04-0.11)	0.06 (0.04-0.11)
**Probability of death for anaemic surgical patients who did not receive transfusions**		0.45 (0.06-0.91)	0.45 (0.06-0.91

**Table 3:** Prevalence of HBsAg and HCV by country.

			TTI prevalence	
			
Country	Reference	Year	HBsAg	Anti HCv
Botswana	[2]	2004	0.05	NA
South Africa	[2]	2004	0.05	NA
Kenya	[2]	2004	0.042	NA
	[39]	1999	0.039	0.018
Tanzania	[40]	2006	0.048	NA
	[41]	2006	0.088	0.015
	[42]	2007	0.053	0.055

NA: not available

### Probabilities

The probability of a surgical patient reaching the transfusion trigger and thus requiring a blood transfusion without receiving TXA (mean probability: 0.66, range: 0-1) was obtained from Davies *et al*. [8]. According to a recent systematic review conducted by Henry *et al*. [4] the relative risk of requiring a blood transfusion following TXA for a surgical patient is 0.61 (CI 95%: 0.54-0.69). Thus, the probability of requiring a blood transfusion after TXA administration (0.40) was estimated by multiplying the baseline risk by the relative risk.

Due to the low rate of voluntary blood donations, less than 52% of the blood requirement was available to be transfused in the WHO African Region [2]. Since there are no published data on the likelihood of adult surgical patients receiving a blood transfusion or on the demand and supply of units of blood during surgery in the four countries considered in the study, the probability of being transfused in a setting where TXA is not routinely used was based on the WHO's recommendations [9,10]. According to WHO [9], a blood supply of between 10-20 whole blood units per 1,000 population will satisfy baseline clinical demand [6,9,10]. Thus, it is assumed that if the volume of blood donated (v) exceeds 0.01 times the population (p), there is no shortage of blood and all surgical patients requiring a transfusion will receive one, otherwise a shortage is assumed and only a proportion of patients will be transfused.

With routine administration of TXA, in a situation of blood shortage, the probability of receiving a blood transfusion, for patients who reach the transfusion trigger, increases because some patients receiving TXA may no longer require a transfusion or will need fewer units thus more units will be available across the proportion of the population that needs transfusions. Thus, the probability of receiving a transfusion (given a shortage of blood) becomes:

Where *m *represents mean number of units transfused in the absence of TXA and *n *is the mean number of units transfused given routine administration of TXA. According to Davies et al. [8] the mean number of units transfused without TXA in elective surgery patients (all types of surgery) having an allogenic blood transfusion, *m*, is 3.13 (2.52-3.73). Thus, the mean number of units required by a patient who received TXA was estimated by subtracting from *m *the estimated reduction in the volume of blood transfused (-1.12 95%CI; -1.59 -0.64)[11].

If patients require on average 3/4 as much blood following TXA, then 4/3 times as many can be transfused using a fixed total number of units. R is the relative risk of a surgical patient requiring a transfusion given routine administration of TXA (0.61 95%CI: 0.54-0.69) [4]. Thus, if half as many require transfusion following TXA, the proportion that can be transfused doubles. The probabilities of being transfused with and without TXA are reported by country in Table 2. In Botswana and South Africa, where the donation rate is relatively high, the administration of TXA does not increase the likelihood of being transfused. However, in countries such as Tanzania and Kenya, which have very low donation rates, the probability more than doubled (Table 1 and 2)[6].

No studies were found that reported the probability of death among elective surgical patients in Africa. According to the meta-analysis of international studies conducted by Davies *et al*. [8] the probability of death for elective surgical patients in high income countries (HICs) is 0.03 (95% CI 0.00-0.21). In order to account for the higher underlying mortality rate of the SSA region, the HICs surgery mortality rate was adjusted according to the following equation:

The estimated probability of dying in SSA is 0.06 [8,12]. In a one way sensitivity analysis, this value was assumed to range between 0.04 and 0.11, with the lower figure representing the underlying probability of dying in sub-Saharan Africa, and the upper figure calculated using the above mentioned formula and Davies' upper estimate [8].

Several studies conducted in SSA have shown that between 16 to 71% of deaths from maternal haemorrhage are due to lack of blood [13]. Nevertheless, no studies were found on death rates of surgical patients in Africa who did not receive clinically indicated blood transfusions. The only data available in the literature come from a cohort study of adults admitted for surgery in the US who refused blood transfusions for religious reasons [14]. This study estimated that the odds of death for a patient with a postoperative Hb of ≤8 g/dl increased 2.5 times (95% CI: 1.9-3.2) for each gram decrease in haemoglobin[14]. In SSA due to the high risk of infections and the lack of blood, patients are usually transfused when their haemoglobin level (Hb) is 5 g/dl or below. Using the relationship estimated by Carson *et al*. [14] a surgical patient in SSA who does not receive a necessary blood transfusion has an estimated probability of dying of 0.45% (95% CI:0.0 to 0.91).

The per-unit risk of HIV, HBV and HCV infections in the four target countries was obtained using a risk model developed by Jayaraman *et al*. [15] where the probability of being HIV, HBV and HCV infected per single unit of blood in each country was estimated according to the formula:

The risk of an infected unit entering the blood supply is estimated using prevalence of infection in donors, screening coverage and test sensitivity. The infectivity risk is the probability of developing HIV, HBV or HCV after receipt of a contaminated unit of blood. The risk of a susceptible person receiving a blood transfusion is dependent on the prevalence of infection in the recipient population.

Assuming that each unit of blood transfused comes from a different donor, administering TXA would reduce both the units of blood required per transfusion and the probability *P*_t _of acquiring HIV, HBV and HCV infection through a blood transfusion [4,16].

Where *p *is the probability of being HIV, HBV or HCV infected *per unit *and *u *is the number of units transfused with and without TXA [16]. The average number of units transfused (3.13 units), when TXA is not given, was taken from the study conducted by Davies *et al*. [8] while the number of units transfused, when TXA is given (2.01), was calculated using Henry et al. [4] results on the effectiveness of TXA in reducing volume of blood transfusion. The HIV prevalence in donors was obtained from the PEPFAR latest release [6]. Data on the HBV and HCV prevalence among blood donors was obtained from a systematic literature review. In Tanzania and Kenya, where more than one estimate of HCV and HBV prevalence was available from the literature, an average of the available values was used to populate the model (Table 3). No data regarding the HCV prevalence in both South Africa and Botswana were retrieved.

### Costs

All costs are reported in 2007 International dollars ($). Two cost items are considered in the analysis: the cost of blood transfusions and the cost of giving TXA.

The cost of providing a unit of blood in Africa from a health and social service perspective depends on the type of system established to provide blood transfusions[17]. Where centralised transfusion services have been established the final cost of producing one unit of blood is higher than for a hospital based service because of the higher cost of recruiting, screening and distributing blood to individual hospitals throughout the country [17]. It has been estimated that costs associated with donor recruitment account for half of the budget of centralised transfusion services[17]. In a hospital based service, on the other hand, the cost of recruiting donors is shifted to the families of the patient that donate the blood or purchase it on the black market [17]. The cost of one unit of blood in Kenya, which relies on a hospital-based service, was assumed to be $15.60 (in 2007 prices) [18,19] because of a lack of country-specific data. A cost of $57.10 was assumed for Tanzania, South Africa and Botswana as these three countries have successfully introduced centralised blood transfusion services with 100% of voluntary donations [1,17,18].

Administering TXA to elective surgical patients is an inexpensive and easy intervention[20]. The time required to administer TXA and observe the patient is short (maximum 15 minutes) and no additional training is required to administer the drug (IV administration is a routine procedure for qualified nurses). Also the supplies required to administer TXA (e.g. 10 mL syringe, 100 ml bag of saline, large gauge needle) are likely to be available and affordable even in limited resource setting. Thus, the assumed costs in the present analysis were $2 and $3 for TXA administration and supplies respectively. The main cost item of the intervention is the drug cost per ampoule of TXA. The cost of TXA might vary by country and by producer [20-22]. Also the dosage needed to prevent fibrinolysis is not well established[4,23]. Horrow *et al*. [24] observed that a dose of 10 mg/kg of TXA followed by 1 mg/kg/hour is effective in decreasing bleeding among surgical patients and that larger doses did not provide any additional haemostatic benefit. As a result, a fixed dose of 2 gram intravenously infused was assumed. In previous trials, it has been observed that this dosage would be efficacious for both larger patients (>100 kg) but also safe in smaller patients (<50 kg) without adverse events[25].

Overhead costs associated with storage, distribution, and inventories were assumed to be zero. TXA is thermally stable and does not require specific storage conditions. Thus, storage and distribution costs per intervention are negligible [5].

The global cost of TXA (Cyklokapron^® ^Pfizer) was obtained from the British National Formulary and converted to 2007 $ using the purchasing power parity exchange rate [26,27].

The overall cost of administering a dose of TXA is estimated to be $13 (made up of a drug cost of $8, staff time $2 and supply cost $3).

### Handling of uncertainty

Univariate deterministic analyses were performed to investigate the impact of selected model parameters on the cost effectiveness of TXA in each of the four countries. As the probability of requiring a blood transfusion without TXA can vary according to the type of surgery, the age of the patient and the adoption of a restrictive transfusion trigger, a broad range of values between 0 and 1 were explored [8]. The relative risk of requiring a blood transfusion with TXA versus no TXA was varied between 0.54 and 0.69 following Henry et al. [11]. The probability of death for a surgical patient requiring and not receiving a blood transfusion was estimated to range between 0.75 (assuming that the patient has a Hb value as low as 3 g/dl) and 0.15 (for patients who are moderately anaemic, Hb = 7 g/dl)[14]. One way sensitivity analysis was also performed to explore how cost effectiveness changed according to the probability of death for a surgical patient (0.04-0.11) and for the number of units transfused to a surgical patient without TXA (2.52-3.73) [8].

In order to account for the potential differences in TXA prices across countries, we use a range of values obtained from the published literature to explore the cost-effectiveness of TXA for different TXA prices in a one way deterministic analysis [28]. The lowest price considered $3.13 (2007 prices), came from a study conducted in Spain that estimated the effectiveness of TXA administration during total knee arthroplasty. The highest value, $44 (2007 prices) was retrieved from an American study on the use of antifibrinolytic agents in surgery for congenital heart disease [22,29]. The cost of a unit of blood was assumed to range between $15.60 (cost of a unit of blood in a hospital based blood system in Africa) and $262 (cost of a unit of red cells in high income countries)[19,30].

A further sensitivity analysis assuming no blood shortage in any the four countries was performed to investigate the effectiveness of TXA in preventing blood borne infection.

A probabilistic approach was adopted in order to assess the impact of the uncertainty more accurately. The beta, gamma and lognormal distributions were chosen for probability, cost and relative risk parameters respectively, following the suggestions of Briggs *et al*. [31]. Monte Carlo simulations were conducted to generate 1,000 samples from the parameter probability distributions [31]. The incremental cost (ΔC), incremental effectiveness (ΔE) and the incremental net benefit of TXA versus no TXA were calculated for each of the Monte Carlo simulations according to the following formula:[31]

Where *λ *is the willingness to pay for a unit change in the outcome (e.g. lives saved). Cost-Effectiveness Acceptability Curves (CEACs) show the probability that the intervention is cost effective, given the range of monetary value the policy maker is willing to pay for a particular unit change in the outcome [31]. CEACs plot the proportion of simulations for which the incremental net benefit of giving TXA versus no TXA is greater than zero (the intervention is cost effective) for a willingness to pay range of $0 to $1,000.

## Results

### Base case analysis

The effectiveness of the intervention varies across countries and depends on the probability of receiving blood with and without the routine use of TXA (Table 2). The overall number of lives saved with TXA versus no TXA is given by the difference in the number of deaths with and without TXA per 1,000 patients. The number of deaths with and without TXA is the is the sum of three elements: deaths of those patients who need a blood transfusion and do not receive it, deaths of patients who need a blood transfusion and receive one, and deaths of patients who did not need a transfusion (die from surgery-related conditions). In Botswana and South Africa, where every patient who needs blood is transfused, the administration of TXA is not lifesaving. In Kenya, where the probability of receiving a blood transfusion despite receiving TXA is 33%, the administration of TXA saves 150 lives per 1,000 patients compared to the do-nothing scenario (Table 4). In Tanzania, TXA is also life saving but the effect is slightly lower with 140 lives saved. According to the present model TXA does not prevent blood-borne viral infections in countries where there is a blood shortage since any blood saved from giving TXA is reallocated to other patients. However, giving TXA in countries where blood is not tested for all viral infections can avert new cases of blood-borne infections. In Botswana, the administration of TXA can avert one HIV case and four HBV infections per 1,000 patients since TXA reduces both the total number of blood transfusions administered in the country and the probability of being infected per blood transfusion. TXA did not prevent HIV in South Africa because the probability of acquiring HIV is very low, less than 1%, even if patients do not receive TXA. However, based on our model, the use of TXA can prevent one case of HBV per 1,000 patients in this setting.

**Table 4:** Cost per life saved per 1,000 surgical patients

Country	**Incremental cost**^**a**^	Lives saved with TXA	Incremental cost per life saved
Botswana	-$59,000^b^	0	Dominant strategy
Kenya	$13,000^b^	150	$87
South Africa	-$59,000^b^	0	Dominant strategy
Tanzania	$13,000^b^	140	$93

**a **All costs are in 2007 prices

**b **rounded to the nearest thousand

The incremental cost of administering TXA in countries where there is blood shortage is $13,000 for 1,000 patients (Table 4). Thus, the estimated incremental cost per life saved is $87 and $93 for Kenya and Tanzania respectively. However, in South Africa and Botswana, where adequate availability of blood ensures access to transfusion for every surgical patient, use of TXA could save $59,000 per 1,000 patients since a lower number of transfusions will be performed (Table 4).

### Sensitivity analysis assuming no shortage of blood

Assuming no shortage of blood in Tanzania, the country with the highest percentage of HIV seropositive blood donations two HIV infections, five HBV infections and thirty-four HCV infections are averted per 1,000 surgical patients who received TXA compared with the no TXA scenario. In Kenya, if blood was available for all patients, the administration of TXA would result in the prevention of four HBV infections and eighteen HCV infections per 1,000 surgical patients.

### One-Way sensitivity analyses

Many parameters used in the model were based on assumptions, or were calculated through equations, using the limited data available from literature. Since these parameters can vary both between and within countries, we performed extensive one-way sensitivity analyses to examine how the cost-effectiveness of TXA is affected by changes in the input values (Table 5).

**Table 5:** One way sensitivity analyses results

	Kenya	Tanzania	Botswana	South Africa
**Probability of requiring a transfusion without TXA**				
0%	$13,000^a^	$13,000^a^	$13,000^a^	$13,000^a^
100%	$58^b^	$63^b^	-$97,000^a^	-$97,000^a^
				
**Probability of death for a patient not receiving transfusion**				
75%	$380^b^	$416^b^	-$59,000^a^	-$59,000^a^
15%	$50^b^	$53^b^	-$59,000^a^	-$59,000^a^
				
**Cost of TXA**				
$3.13	$54^b^	$59^b^	-$64,000^a^	-$64,000^a^
$44	$327^b^	$350^b^	-$23,000^a^	-$23,000^a^
				
**Cost of one unit of blood**				
$15.6	$87^b^	$93^b^	-$6,000^a^	-$6,000^a^
$262	$87^b^	$93^b^	-$316,000^a^	-$316,000^a^
				
**Probability of death for a surgery patient in SSA**				
11%	$100^b^	$108b	-$59,000^a^	-$59,000^a^
4%	$83^b^	$88^b^	-$59,000^a^	-$59,000^a^
				
**RR of requiring transfusion with TXA vs. no TXA**				
0.54	$78^b^	$88^b^	-$64,000^a^	-$64,000^a^
0.69	$102^b^	$172^b^	-$52,000^a^	-$52,000^a^
				
**Number of units transfused without TXA**				
2.52	$105^b^	$110^b^	$-36,000^a^	$-36,000^a^
3.73	$74^b^	$81^b^	$-81,000^a^	$-81,000^a^

**a **rounded to the nearest thousand

**b **Incremental cost per life saved

As shown in Table 5, the results for Botswana and South Africa are identical. In both the countries, all patients needing a blood transfusion receive one. In all four countries the probability of requiring a blood transfusion without TXA has the greatest impact on the cost effectiveness of TXA. If no-one requires a transfusion, giving TXA increases costs by $13,000 (the cost of administering TXA to 1,000 patients). For the case in which everyone requires a transfusion, TXA is cost saving in Botswana and South Africa (-$97,000 per 1,000 patients) and life saving in Tanzania and Kenya (incremental cost per additional life saved $63 and $58).

Changes in the probability of death for an anaemic patient who does not receive a blood transfusion also affects model outcomes. The higher the probability of death for those not receiving a transfusion, the lower the incremental cost per life saved of administering TXA on a routine basis to surgical patients. Assuming that patients not receiving transfusions are moderately anaemic (7 dl/g), the incremental cost per life saved of administering TXA is $380 and $416 for Kenya and Tanzania respectively. However, if all the patients requiring transfusion are severely anaemic (4-3 g/dl), this value decreases to approximately $50 per life saved [14].

The incremental cost per life saved is also sensitive to the cost of TXA. Assuming a cost of $3.13, the incremental cost per life saved is $54 in Kenya and $59 in Tanzania. With a TXA cost of $44, the cost per life saved increases to $327 and $350. In both Botswana and South Africa, TXA is always cost saving regardless of the price of the intervention. In those countries where there is a shortage of blood, variations in the cost of the blood do not affect the cost effectiveness of TXA since the same amount of blood will be transfused overall.

The cost-effectiveness of administering TXA is also relatively sensitive to changes in the probability of death for surgical patients in SSA. Holding other variables constant, the incremental cost per life saved increases as the probability of death among surgical patients increases. Ranging from $83 and $88 per life saved, assuming a probability of death of 0.04%, to $100 and $108 assuming a probability of death of 0.11%. Both the Relative Risk reduction of requiring a blood transfusion with TXA and the number of units transfused without TXA affect the model results. However, independent of these two parameter changes, TXA remains very cost effective in Tanzania and Kenya and cost saving in Botswana and South Africa.

### Probabilistic sensitivity analysis

**Figure 3:**
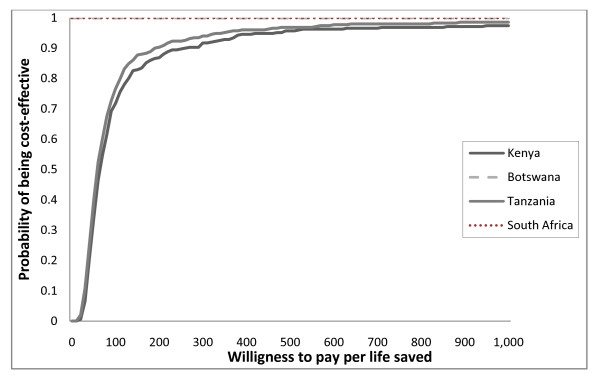
**Cost-effectiveness Acceptability curves showing the probability that administering TXA is cost effective in the four countries**.

Figure 3 presents the results of the probabilistic sensitivity analysis. The vertical axis shows the probability of the intervention of being cost-effective given the willingness to pay (WTP) per life saved reported on the horizontal axis. The probability that the intervention is cost saving is indicated by the point where the CEAC cuts the vertical axis since a zero value for *λ *implies that the policy maker places no value on lives saved. Thus in Botswana and South Africa, routine use of TXA is expected to be cost-saving for all of the potential combinations of parameters considered in this analysis. Whereas, in Kenya and Tanzania, the routine use of TXA has a zero probability of being cost effective when the WTP per life saved is zero; this value rises to 0.7 for a WTP per life saved higher than $100 and reaches a plateau above 0.9 for a WTP higher than $400.

## Discussion

The routine administration of TXA in bleeding elective surgical patients could be life saving in countries such as Kenya and Tanzania where there is a shortage of blood because more blood will be available for those who need it. In countries where blood is readily available, such as South Africa and Botswana, the use of TXA is likely to be cost saving because the savings from reducing the number of blood transfusions needed exceed the cost of administering TXA routinely to bleeding surgical patients. In addition, where there is no blood shortage, the administration of TXA decreases the risk of transfusion-transmitted viral infections because fewer units of blood will be transfused. Therefore, independent of the cost-effectiveness threshold for adopting a health care intervention, administering TXA is a dominant strategy in countries where blood transfusions are readily available.

There is reliable evidence from randomised controlled trials that the administration of TXA to bleeding elective surgical patients reduces the need for blood transfusions and reduces the amount of blood transfused [4]. Kenya, Botswana, Tanzania and South Africa were selected in order to evaluate how the cost effectiveness of administering TXA to bleeding surgical patients varies according to country-specific circumstances, specifically different blood donation rates and HIV seroprevalence [6]. Even in countries where there is a shortage of blood, TXA is a highly cost-effective intervention. According to the Commission of Macroeconomics and Health [32], in the context of developing countries a very cost effective intervention would avert one disability adjusted life year (DALY) for less than the average per capita income for a given country or region. The estimated cost per life saved is $93 and $83 in Tanzania and Kenya respectively. Thus, assuming that a surgical patient whose life was saved due to TXA administration survives for even one year (in perfect health) the cost per DALY averted resulting from using TXA would be well below the average per capita income of Tanzania ($400) and Kenya ($680) [33].

However, there are important limitations to the data used in the model and these need to be taken into account when interpreting the findings. Several model parameters were not available in the literature and were estimated indirectly through equations which made several strong assumptions. For example, it was assumed that the blood savings arising from use of TXA would be re-distributed among other surgical patients and not used to treat other patient groups requiring blood transfusions. Also the model did not account for intra-country variation in healthcare infrastructure. In rural areas for instance, the probability of death for a surgical patient and the probability of being HIV, HBV and HCV infected may be higher as both qualified personnel and reagents for blood screening are less likely to be available. Another potential limitation is that the risk of being transfused and the risk reduction driven by TXA were taken from studies conducted in developed countries [4,8]. In particular, since the rate of preoperative anaemia among surgical patients in SSA is higher than in HICs, it is likely that the present study is underestimating the risk of being transfused and so the potential benefit of administering TXA. According to the meta-analysis conducted by Henry *et al*. [4] TXA there is no reliable evidence that TXA is associated with an increased risk of adverse events such as myocardial infarction (RR 0.96 95%CI 0.48 to1.90), increased risk of stroke (RR 1.25 95%CI 0.47 to 3.31) and thrombotic (RR 0.77 95%CI 0.37 to1.61) events in HICs. However, it is unclear if this may also be the case in elective surgical patients in SSA. Cost estimates could also have been a source of error in our model.

The analysis did not account for the potential cost savings for surgical patients arising from TXA administration. For example, in those African countries, such as Mozambique, where the cost for blood transfusion services is recovered from the beneficiaries of the transfusion, TXA administration would reduce the financial burden for the patients [34].

The epidemiological transition in Africa is moving the demand for surgery to conditions similar to those observed in developed countries. Especially in the urban areas, where the change in life expectancy and health behaviours occur at a faster pace, the higher incidence of non communicable diseases will contribute to an increased demand for elective surgical procedures. Ischemic heart disease, which is the most common cause of cardiac surgery, now ranks 8^th ^among the causes of death in SSA and is already the leading cause of death among the elderly (>60 years) [35]. For example, it was estimated that ischemic heart disease alone accounted for 13660 deaths in Kenya and 27013 in South Africa in 2002 [35]. Another cause of elective surgery (e.g. hip replacement), rheumatoid arthritis, once considered a rarity in SSA, has now become a common disease in many countries [36].

This study evaluates the cost-effectiveness of administering TXA among elective surgical patients in general without distinguishing between different types of surgery (e.g. cardiac surgery, orthopaedic surgery). This is justified since according to the meta-analysis conducted by Henry et al. [11] TXA shows similar effectiveness in reducing both the risk and the volume of blood transfused across all the types of elective surgery. In order to account for the difference in the risk of receiving a blood transfusion (with and without TXA) between types of surgery and between countries, extensive one way sensitivity analyses have been performed.

As this was a simulation study, the data to populate the model came from different sources and settings, which could have affected the parameter estimates. It is possible that TXA may also reduce mortality (RR 0.60, 95% CI: 0.32-1.12) and the risk of re-operation for bleeding (RR 0.67, 95% CI: 0.41-1.09) [11]. Although both these outcomes were not statistically significant, it would be important to consider them in future studies evaluating TXA cost effectiveness. Finally, as no data were found for the HCV prevalence among the donor population in both South Africa and Botswana, it was not possible to estimate whether in these two countries administration of TXA would lead to a reduction in the number of HCV infections transmitted through blood transfusions.

According to Ozgediz and Riviello [37], although surgical conditions account for 11% of the global burden of diseases, with 25 million disability-adjusted life years in Africa, surgical procedures are "neglected diseases" in LMICs and in particular in sub-Saharan Africa [37,38]. This study has shown that the routine administration of TXA could be a very cost effective intervention for reducing both the cost and the risks associated with surgical procedures requiring blood transfusions in sub-Saharan Africa [37,38]. It has been demonstrated that TXA could be potentially lifesaving in those African countries where there is blood shortage. Moreover, it can also reduce cost and prevent some blood borne infections where blood is readily available.

## Competing interests

The authors declare that they have no competing interests.

## Authors' contributions

CG carried out the analysis and wrote the draft of the manuscript. JC help in designing and conducting the analysis, contributed in revising the manuscript and interpreting the main results findings. SJ helped with conducting the analysis and revising the manuscript. IR helped design of the model by providing epidemiological inputs and revised the manuscript. PP helped with interpretation of results and critically revised the manuscript. HS helped in the design of the study and contributed to critically revise the manuscript. All authors read and approved the final manuscript.
